# Effect of Myoinositol and Antioxidants on Sperm Quality in Men with Metabolic Syndrome

**DOI:** 10.1155/2016/1674950

**Published:** 2016-09-26

**Authors:** Mario Montanino Oliva, Elisa Minutolo, Assunta Lippa, Paola Iaconianni, Alberto Vaiarelli

**Affiliations:** ^1^Department of Obstetrics & Gynecology, Santo Spirito Hospital, 00193 Rome, Italy; ^2^Altamedica IVF Unit, 00198 Rome, Italy; ^3^Reproductive Medicine Unit, University Hospital, 98122 Messina, Italy

## Abstract

This prospective longitudinal study investigated the effects of a dietary supplement in patients affected by reduced sperm motility (asthenospermic males) with metabolic syndrome. The product tested was Andrositol®, which contains myoinositol (MI) as principal compound, in association with other molecules, and the parameters evaluated were semen characteristics as well as hormone and metabolic profiles. The inclusion criteria were subjects aged over 18 years, with asthenospermia and metabolic syndrome. The exclusion criteria were presence of cryptorchidism, varicocele, and prostatitis. For this study, 45 males who had such features were enrolled. Their selection was made according to the 2010 World Health Organization (WHO) criteria (5th Edition) for the Evaluation of Human Semen. Hormone and metabolic profiles and semen parameters were assessed at the beginning of the study and after three months of treatment with Andrositol. The differences between the values before and after the supplementation were found statistically significant. Andrositol normalized the metabolic profile of these patients, improving their insulin sensitivity. Moreover, testosterone levels were increased and the semen characteristics, such as sperm concentration, motility, and morphology, highly improved. In conclusion, the association of MI with other molecules (micronutrients and vitamins) could be an effective therapy for metabolic disorders, as well as hormonal and spermatic changes responsible for male infertility.

## 1. Introduction

Myoinositol (MI) is a sugar-like molecule and it is one of the precursors for the synthesis of phosphatidylinositol polyphosphates (PIPs), key biomolecules belonging to the signal transduction system of several cellular functions [1]. MI exerts a variety of clinical implications, in respect to several pathological conditions, such as metabolic syndrome (MS) and other diseases associated with it [2, 3]. MS is a complex disorder, characterized by alterations in carbohydrate metabolism, obesity, systemic arterial hypertension, and dyslipidemia [4]. These alterations may affect different neuroendocrine axis controlled by hypothalamus and pituitary [4–6].

Recently, a bidirectional interaction between MS and asthenospermia has been proved [7]. In asthenospermia, the proportion of motile sperms is below the World Health Organization's (WHO) standard leading to infertility [8]. High reactive oxygen species (ROS) levels in the semen might be an etiologic factor for male infertility [9]. It is estimated that 25% of infertile men possess high levels of semen ROS. A fertile man does not have high levels of semen ROS instead [10, 11]. ROS are needed for capacitation, acrosome reaction, and ultimately fertilization [12]. However, their uncontrolled production is dangerous for a variety of biomolecules such as lipids, amino acids, carbohydrates, protein, and DNA. High ROS levels negatively affect sperm function [13] due to DNA damage [14, 15]. Furthermore, they reduce sperm motility [16] and impair membrane integrity [17, 18]. Several therapies attempted to counteract the conditions leading to infertility; unfortunately, the totality were found ineffective, except the treatment with some antioxidants [19, 20]. MI supplementation was demonstrated to be really effective in improving semen parameters* in vitro* [21, 22] in oligoteratoasthenozoospermic (OAT) patients.

The present study attempts to determine, for the first time, the effects of a dietary supplement containing MI, selenium, and L-arginine in asthenospermic patients affected by MS. The authors evaluated the effects of this administration on semen parameters, as well as on hormone and metabolic profiles.

## 2. Patients and Methods

### 2.1. Patients

This is a prospective longitudinal study of asthenospermic males with MS, under treatment at Altamedica IVF Unit, Rome, Italy. All participants have signed an informed consent form. The inclusion criteria were subjects aged over 18 years, with asthenospermia and MS. The exclusion criteria were presence of cryptorchidism, varicocele, and prostatitis. Overall, 45 males were enrolled from January to April, 2011. Asthenospermia was defined according to the 2010 World Health Organization criteria (5th Edition) for the Evaluation of Human Semen. The following parameters, established by the National Cholesterol Education Program, were used to assess whether the subjects were affected by MS: fasting plasma glucose ≥ 100 mg/dL or diagnosis of diabetes; waist circumference > 102 cm in males; blood pressure ≥ 130/85 mm Hg; triglycerides ≥ 150 mg/dL; HDL cholesterol < 40 mg/dL in males.

The medical histories of all patients were taken into consideration and physical examinations were conducted by the same physicians.

Hormone and metabolic profiles as well as semen parameters were evaluated in the relevant study at the beginning and after three months of therapy. The patients were treated by a dietary supplement administered twice a day containing 1 g MI, 30 mg L-carnitine, L-arginine and vitamin E, 55 *μ*g selenium, and 200 *μ*g folic acid (Andrositol, Lo.Li. Pharma s.r.l., Rome).

### 2.2. Samples

In order to determinate metabolic and hormonal profiles, the semen and blood samples were obtained from all patients before and at the end of treatment. The semen samples, obtained by masturbation after 3 to 5 days of sexual abstinence, were analysed immediately after complete liquefaction. Each patient was asked to provide three samples, taken up in different days, with the purpose to reduce the variability due to the use of drugs and alcohol or the presence of fever in the days before the test.

Sperm features were evaluated by the same examiner, according to the World Health Organization guidelines (World Health Organization, 2010).

The homeostasis model for insulin resistance (HOMA) index was calculated as the product of the fasting plasma insulin (mIU/mL) and glucose (mmol/L) concentrations divided by 22.5 [23]. Waist circumference (WC), body mass index (BMI), and triglycerides (TG) were determined prior to and after MI supplementation.

Plasma luteinizing hormone (LH), follicle-stimulating hormone (FSH), estradiol (E2), sex hormone-binding globulin (SHBG), free testosterone, and testosterone (T) levels were measured by radioimmunoassay (RIA). These hormones may contribute to changing sperm concentration, total motility, and morphology [24, 25].

### 2.3. Statistical Analysis

The results are presented as mean ± standard deviation. The differences in variables before and after MI supplementation were statistically analysed with Student's paired *t*-test. *P* < 0.05 was considered statistically significant. GraphPad Prism software (GraphPad Software, Inc., La Jolla, CA, USA) was employed for the data analysis.

## 3. Results

Table 1 shows patients' metabolic and hormone profiles. Although statistically significant differences were not observed in BMI, waist circumference, or triglyceride levels before and after the treatment with Andrositol for three months, the HOMA index significantly decreased after the therapy (*P* < 0.001).

In regard of hormone profile, only FSH levels were not affected by the supplementation, whilst plasma, E2, and SHBG levels decreased significantly after the treatment (*P* < 0.01 and *P* < 0.001, resp.). Following the treatment, authors observed that a statistically significant increase in LH levels (*P* < 0.01), as well as in free (*P* < 0.001) and total testosterone levels (*P* < 0.02), occurred.  Table 2 shows the semen characteristics evaluated before and after Andrositol administration.

After the treatment, all sperm characteristics were significantly improved (*P* < 0.001 for sperm concentration, motility, and morphology).

## 4. Discussion

Herein, the authors have determined the effects of a treatment for three months by using a dietary supplement containing MI, in association with other micronutrients and vitamins on metabolic and hormone profiles and on semen parameters of asthenospermic patients with MS.

MS is a combination of disorders, including obesity, that can increase the risk of developing cardiovascular disease and diabetes [26, 27]. It is largely known that components of MS induce reproductive axis disorders [27], though this strong link remains still unclear [28].

Insulin resistance is likely to be responsible, along with adipose tissue mediated signs, for the regulation of gonadal function, causing changes in hormone and proinflammatory cytokines levels [4, 29–31].

Male infertility is frequently due to asthenospermia which ensues a reduction in sperm motility [32].

Furthermore, this condition is generally associated with decreased T, plus elevated E2, FSH, and LH levels [25].

To date, this is the first* in vivo* study that focuses on this topic. Nevertheless, several evidences of MI clinical efficacy in women with PCOS [33–35] and in postmenopausal women with MS are available in literature [12, 14].

The administration of this dietary supplement normalized the metabolic profile of asthenospermic patients with MS, increasing their insulin sensitivity without significant changes in BMI, WC, and triglycerides levels. Moreover, in these subjects, the treatment increased T levels and significantly improved semen characteristics.

It is noteworthy that reduced T levels have been associated with MS and particularly obesity [36]. We may speculate that such decrease is caused by the increased transformation of androgens to estrogens (e.g., E2), by means of aromatization in the peripheral deposits of fat [37, 38]. This imbalance, together with elevated SHBG levels, ends up causing male infertility. It is a consequence of a reduction in sperm concentration and motility, as well as an alteration of sperm morphology [25, 39]. It is interesting to note that the decreased T concentrations constitute in men one of the predicting factors for the onset of insulin resistance, type 2 diabetes, and MS [40, 41].

A replacement therapy with T may improve this condition in these patients; however, it is not completely effective for the treatment of infertility in men [42, 43].

Therefore, new drugs and molecules that increase insulin sensitivity, raising T levels at the same time, are desirable.

Recent studies [44] suggested the involvement of inositols in spermatozoa maturation as well as in their migration from the epididymis. It is interesting to notice that MI concentration is significantly higher in tubules than in serum and other organs [44]. In line with these observations, low MI levels within epididymis and in seminal fluid are associated with reduced fertility [45]. Moreover, MI plays a role in the chemiotaxis and human sperm thermotaxis through the activation of PLC. It results in production of InsP_3_ and calcium channels opening leading to an increase in Ca^2+^ intracellular concentrations in the flagellum [46]. A recent study highlighted that MI improves OAT samples quality by removing amorphous material. That is likely to be responsible for the high viscosity of seminal fluid [32].

Our clinical study has demonstrated its effectiveness for MS as well as ameliorating the hormonal and spermatic conditions which imply male infertility. As shown, patients with asthenospermia and MS definitely improved their condition after MI use.

The association of selenium and arginine with MI appears particularly interesting. In fact, selenium is a micronutrient important for the male gonad and the process of male reproduction [47, 48]. This element exerts an antioxidant activity mediated by several selenoproteins involved in crucial physiological processes (reproduction, aging, immunity, etc.) [49]. The phospholipid hydroperoxide glutathione peroxidase (PHGPx) plays a pivotal function for male fertility by preserving the cells, undergoing rapid division from oxidative stress as well as stimulating important processes of differentiation [50]. It was demonstrated that PHGPx is necessary for stabilizing the sperm mitochondrial collar and protecting the phospholipids of the germ cell membrane from peroxidation [51].

Also a deficiency in L-arginine content is harmful for male fertility since this amino acid is strongly involved in the process of sperm formation [52]. Reduced levels of L-arginine alter sperm metabolism with the consequence of a decreased motility and spermatogenesis [53]. According to these observations, infertile patients show an increased sperm count and motility when they are treated with L-arginine which does not carry side effects [54]. The relevance of this amino acid for sperm was demonstrated* in vitro* with humans, rabbits, and goats [55–57]. In particular, it prevents the peroxidation of sperm membrane lipids subjected to different stress conditions [58, 59].

Moreover, L-arginine is essential to relieve constricted arteries due to its role in generating a mediator called nitric oxide. It was shown that the administration of this amino acid helps the artery dilatation, increasing blood volume [60].

Overall, these clinical results lead us to deem that the success of the therapy with Andrositol could be mainly due to the association of MI with selenium and L-arginine. It is likely that the antioxidant role of these last two molecules has been important for the improvement of sperm parameters. On the other hand, MI may have helped to balance the hormonal and metabolic parameters, as it acts as second messenger regulating the activities of several hormones such as FSH, TSH, and insulin [61]. In conclusion, this dietary supplement has significantly improved the clinical condition of the asthenospermic patients with MS; therefore, its use should be encouraged.

There are several limitations to the present study, such as lack of controls, limited number of patients, brief treatment period, and unsegmented group of subjects with MS. Therefore, in follow-up, larger prospective randomized case-control studies are needed to elucidate the role and effects exerted by MI, selenium, and L-arginine in asthenospermic patients with different clinical presentations of MS.

## Figures and Tables

**Table 1:** Metabolic and hormone profiles before and after treatment.

Analysis	Before	After	*P *value
*Metabolic profile*			
BMI (kg/cm^2^)	28.1 ± 3.5	27.0 ± 3.1	NS
WC (cm)	107.1 ± 4.2	105.3 ± 3.3	NS
Triglycerides (mg/dL)	175.4 ± 12.5	173.2 ± 13.4	NS
HOMA index	2.8 ± 1.2	1.6 ± 0.7	<0.001
SBP	135.3 ± 12.7	128.9 ± 11.0	NS
DBP	91.2 ± 9.2	82.6 ± 9.3	NS
*Hormone profile*			
LH (mIU/mL)	2.5 ± 1.3	3.3 ± 1.2	<0.01
FSH (mIU/mL)	3.4 ± 1.2	3.5 ± 1.1	NS
E2 (pg/mL)	32.4 ± 5.2	20.9 ± 3.3	<0.01
SHBG (nmol/mL)	55.0 ± 4.9	35.8 ± 3.5	<0.001
Free testosterone (pg/mL)	33.0 ± 11.1	47.2 ± 13.0	<0.001
Total testosterone (ng/mL)	2.8 ± 1.2	3.7 ± 1.4	<0.02

Data are mean ± standard deviation. BMI: body mass index; DBP: Diastolic Blood Pressure; E2: estradiol; FSH: follicle-stimulating hormone; HOMA: homeostasis model for insulin resistance; LH: luteinizing hormone; NS: not statistically significant; SBP: Systolic Blood Pressure; SHBG: sex hormone-binding globulin; WC: waist circumference.

**Table 2:** Semen analysis before and after treatment.

Semen parameter	Before	After	*P *value
Concentration (10^6^/mL)	16.2 ± 3.4	20 ± 4.2	<0.001
Motility (%)	39.6 ± 6.1	51.4 ± 7.2	<0.001
Normal morphology (%) (normal)	24.9 ± 2.0	30.1 ± 2.3	<0.001

Data are mean ± standard deviation.

## References

[1] Carlomagno G., Nordio M., Chiu T. T., Unfer V. (2011). Contribution of myo-inositol and melatonin to human reproduction. *European Journal of Obstetrics Gynecology and Reproductive Biology*.

[2] Berridge M. J. (1993). Inositol trisphosphate and calcium signalling. *Nature*.

[3] Divecha N., Irvine R. F. (1995). Phospholipid signaling. *Cell*.

[4] Matos A. F., Moreira R. O., Guedes E. P. (2003). Aspectos neuroendócrinos da síndrome metabólica. *Arquivos Brasileiros de Endocrinologia & Metabologia*.

[5] Carvalheira J. B. C., Saad M. J. A. (2006). Doenças associadas à resistência à insulina/hiperinsulinemia, não incluídas na síndrome metabólica. *Arquivos Brasileiros de Endocrinologia & Metabologia*.

[6] Livingstone C., Collison M. (2002). Sex steroids and insulin resistance. *Clinical Science*.

[7] Goulis D. G., Tarlatzis B. C. (2008). Metabolic syndrome and reproduction: I. Testicular function. *Gynecological Endocrinology*.

[8] Cooper T. G., Noonan E., von Eckardstein S. (2009). World Health Organization reference values for human semen characteristics. *Human Reproduction Update*.

[9] Desai N., Sharma R., Makker K., Sabanegh E., Agarwal A. (2009). Physiologic and pathologic levels of reactive oxygen species in neat semen of infertile men. *Fertility and Sterility*.

[10] Zini A., San Gabriel M., Baazeem A. (2009). Antioxidants and sperm DNA damage: a clinical perspective. *Journal of Assisted Reproduction and Genetics*.

[11] Agarwal A., Nallella K. P., Allamaneni S. S. R., Said T. M. (2004). Role of antioxidants in treatment of male infertility: an overview of the literature. *Reproductive BioMedicine Online*.

[12] Santamaria A., Giordano D., Corrado F. (2012). One-year effects of myo-inositol supplementation in postmenopausal women with metabolic syndrome. *Climacteric*.

[13] Rivlin J., Mendel J., Rubinstein S., Etkovitz N., Breitbart H. (2004). Role of hydrogen peroxide in sperm capacitation and acrosome reaction. *Biology of Reproduction*.

[14] Giordano D., Corrado F., Santamaria A. (2011). Effects of myo-inositol supplementation in postmenopausal women with metabolic syndrome: a perspective, randomized, placebo-controlled study. *Menopause*.

[15] Aitken R. J., De Iuliis G. N., Finnie J. M., Hedges A., McLachlan R. I. (2010). Analysis of the relationships between oxidative stress, DNA damage and sperm vitality in a patient population: development of diagnostic criteria. *Human Reproduction*.

[16] Kao S.-H., Chao H.-T., Chen H.-W., Hwang T. I. S., Liao T.-L., Wei Y.-H. (2008). Increase of oxidative stress in human sperm with lower motility. *Fertility and Sterility*.

[17] Aitken R. J., Clarkson J. S., Fishel S. (1989). Generation of reactive oxygen species, lipid peroxidation, and human sperm function. *Biology of Reproduction*.

[18] Agarwal A., Saleh R. A., Bedaiwy M. A. (2003). Role of reactive oxygen species in the pathophysiology of human reproduction. *Fertility and Sterility*.

[19] Showell M. G., Mackenzie-Proctor R., Brown J., Yazdani A., Stankiewicz M. T., Hart R. J. (2014). Antioxidants for male subfertility. *The Cochrane Database of Systematic Reviews*.

[20] Kumalic S. I., Pinter B. (2014). Review of clinical trials on effects of oral antioxidants on basic semen and other parameters in idiopathic Oligoasthenoteratozoospermia. *BioMed Research International*.

[21] Condorelli R. A., La Vignera S., Di Bari F., Unfer V., Calogero A. E. (2011). Effects of myoinositol on sperm mitochondrial function in-vitro. *European Review for Medical and Pharmacological Sciences*.

[22] Condorelli R. A., La Vignera S., Bellanca S., Vicari E., Calogero A. E. (2012). Myoinositol: does it improve sperm mitochondrial function and sperm motility?. *Urology*.

[23] Matthews D. R., Hosker J. P., Rudenski A. S., Naylor B. A., Treacher D. F., Turner R. C. (1985). Homeostasis model assessment: insulin resistance and *β*-cell function from fasting plasma glucose and insulin concentrations in man. *Diabetologia*.

[24] Cavallini G. (2006). Male idiopathic oligoasthenoteratozoospermia. *Asian Journal of Andrology*.

[25] Robeva R., Sestrimska N., Atanasova I., Mekhandzhiev T., Tomova A., Kumanov F. (2008). Sperm disorder in males with obesity and metabolic syndrome—pilot study. *Akusherstvo I Ginekologiia*.

[26] Michalakis K., Mintziori G., Kaprara A., Tarlatzis B. C., Goulis D. G. (2013). The complex interaction between obesity, metabolic syndrome and reproductive axis: a narrative review. *Metabolism: Clinical and Experimental*.

[27] Caldas A. D. A., Porto A. L., Motta L. D. C. D., Casulari L. A. (2009). Relationship between insulin and hypogonadism in men with metabolic syndrome. *Arquivos Brasileiros de Endocrinologia & Metabologia*.

[28] Lordelo R. A., Mancini M. C., Cercato C., Halpern A. (2007). Eixos hormonais na obesidade: causa ou efeito?. *Arquivos Brasileiros de Endocrinologia & Metabologia*.

[29] Isidori A. M., Caprio M., Strollo F. (1999). Leptin and androgens in male obesity: evidence for leptin contribution to reduced androgen levels. *The Journal of Clinical Endocrinology & Metabolism*.

[30] Magni P., Motta M., Martini L. (2000). Leptin: a possible link between food intake, energy expenditure, and reproductive function. *Regulatory Peptides*.

[31] Jungwirth A., Giwercman A., Tournaye H. (2012). European association of urology working group on male infertility. European Association of Urology guidelines on male infertility: the 2012 update. *European Urology*.

[32] Colone M., Marelli G., Unfer V., Bozzuto G., Molinari A., Stringaro A. (2010). Inositol activity in oligoasthenoteratospermia—an in vitro study. *European Review for Medical and Pharmacological Sciences*.

[33] Unfer V., Carlomagno G., Dante G., Facchinetti F. (2012). Effects of myo-inositol in women with PCOS: a systematic review of randomized controlled trials. *Gynecological Endocrinology*.

[34] Nestler J. E., Jakubowicz D. J., Reamer P., Gunn R. D., Allan G. (1999). Ovulatory and metabolic effects of D-chiro-inositol in the polycystic ovary syndrome. *The New England Journal of Medicine*.

[35] Genazzani A. D., Lanzoni C., Ricchieri F., Jasonni V. M. (2008). Myo-inositol administration positively affects hyperinsulinemia and hormonal parameters in overweight patients with polycystic ovary syndrome. *Gynecological Endocrinology*.

[36] Teerds K. J., de Rooij D. G., Keijer J. (2011). Functional relationship between obesity and male reproduction: from humans to animal models. *Human Reproduction Update*.

[37] Bray G. A. (1997). Obesity and reproduction. *Human Reproduction*.

[38] Foresta C., Di Mambro A., Pagano C., Garolla A., Vettor R., Ferlin A. (2009). Insulin-like factor 3 as a marker of testicular function in obese men. *Clinical Endocrinology*.

[39] Pavlovich C. P., King P., Goldstein M., Schlegel P. N. (2001). Evidence of a treatable endocrinopathy in infertile men. *The Journal of Urology*.

[40] Laaksonen D. E., Niskanen L., Punnonen K. (2004). Testosterone and sex hormone-binding globulin predict the metabolic syndrome and diabetes in middle-aged men. *Diabetes Care*.

[41] Muller M., Grobbee D. E., den Tonkelaar I., Lamberts S. W. J., van der Schouw Y. T. (2005). Endogenous sex hormones and metabolic syndrome in aging men. *The Journal of Clinical Endocrinology & Metabolism*.

[42] Basaria S. (2008). Androgen deprivation therapy, insulin resistance, and cardiovascular mortality: an inconvenient truth. *Journal of Andrology*.

[43] Liu P. Y., Handelsman D. J. (2003). The present and future state of hormonal treatment for male infertility. *Human Reproduction Update*.

[44] Boni R., Gualtieri R., Talevi R., Tosti E. (2007). Calcium and other ion dynamics during gamete maturation and fertilization. *Theriogenology*.

[45] Yeung C.-H., Anapolski M., Setiawan I., Lang F., Cooper T. G. (2004). Effects of putative epididymal osmolytes on sperm volume regulation of fertile and infertile c-ros transgenic mice. *Journal of Andrology*.

[46] Bahat A., Eisenbach M. (2010). Human sperm thermotaxis is mediated by phospholipase C and inositol trisphosphate receptor Ca^2+^ channel. *Biology of Reproduction*.

[47] Shalini S., Bansal M. P. (2005). Role of selenium in regulation of spermatogenesis: involvement of activator protein 1. *BioFactors*.

[48] Ahsan U., Kamran Z., Raza I. (2014). Role of selenium in male reproduction—a review. *Animal Reproduction Science*.

[49] Li J.-L., Li H.-X., Li S., Jiang Z.-H., Xu S.-W., Tang Z.-X. (2011). Selenoprotein W gene expression in the gastrointestinal tract of chicken is affected by dietary selenium. *BioMetals*.

[50] Bauché F., Fouchard M.-H., Jégou B. (1994). Antioxidant system in rat testicular cells. *FEBS Letters*.

[51] Foresta C., Flohé L., Garolla A., Roveri A., Ursini F., Maiorino M. (2002). Male fertility is linked to the selenoprotein phospholipid hydroperoxide glutathione peroxidase. *Biology of Reproduction*.

[52] Miroueh A. (1970). Effect of arginine on oligospermia. *Fertility and Sterility*.

[53] Holt L. E., Albanesi A. A. (1944). Observation of amino acids deficiencies in man. *Transactions of the Association of American Physicians*.

[54] Scibona M., Meschini P., Capparelli S., Pecori C., Rossi P., Fabris G. F. M. (1994). Arginine and male infertility. *Minerva Urologica e Nefrologica*.

[55] Aydin S., Inci O., Alagöl B. (1995). The role of arginine, indomethacin and kallikrein in the treatment of oligoasthenospermia. *International Urology and Nephrology*.

[56] Radany E. W., Atherton R. W., Forrester I. T. (1981). Arginine uptake by rabbit spermatozoa. *Archives of Biochemistry and Biophysics*.

[57] Patel A. B., Srivastava S., Phadke R. S., Govil G. (1998). Arginine activates glycolysis of goat epididymal spermatozoa: an NMR study. *Biophysical Journal*.

[58] Govil G., Phadke R. S., Srivastava S., Ong A. S. H., Packer L. (1992). Physical/chemical studies of vitamin E in membranes. *Lipid-Soluble Antioxidants: Biochemistry and Clinical Applications*.

[59] Srivastava S., Desai P., Coutinho E., Govil G. (2000). Protective effect of L-arginine against lipid peroxidation in goat epididymal spermatozoa. *Physiological Chemistry and Physics and Medical NMR*.

[60] Srivastava S., Desai P., Coutinho E., Govil G. (2006). Mechanism of action of l-arginine on the vitality of spermatozoa is primarily through increased biosynthesis of nitric oxide. *Biology of Reproduction*.

[61] Bizzarri M., Carlomagno G. (2014). Inositol: history of an effective therapy for polycystic ovary syndrome. *European Review for Medical and Pharmacological Sciences*.

